# Complete Genome Sequences of *Weissella cibaria* Strains CMU, CMS1, CMS2, and CMS3 Isolated from Infant Saliva in South Korea

**DOI:** 10.1128/genomeA.01103-17

**Published:** 2017-10-05

**Authors:** Mi-Sun Kang, Ji-Eun Yeu, Jong-Suk Oh, Boo-Ahn Shin, Jin-Hee Kim

**Affiliations:** a Research Institute, Oradentics Co., Ltd., Seoul, South Korea; b Department of Microbiology, School of Medicine, Chonnam National University, Gwangju, South Korea

## Abstract

*Weissella cibaria* strain CMU is used as a commercial oral care probiotic in South Korea. Here, we present the complete genome sequences of four *W. cibaria* strains (CMU, CMS1, CMS2, and CMS3) isolated from the saliva of an infant living in Gwangju, South Korea.

## GENOME ANNOUNCEMENT

*Weissella cibaria* is a short, rod-shaped, Gram-positive, non-spore-forming, nonmotile, heterofermentative, and catalase-negative lactic acid bacterium. This species is widely distributed in human saliva and feces (1–3) and in traditional fermented foods such as kimchi, tarhana, and sourdoughs (4–6). Recently, oral care probiotics have been introduced to substitute conventional treatment for oral diseases, including dental caries, gingivitis, and chronic periodontitis (7). Our research group has shown that *W. cibaria* strains CMU (ÖraCMU), CMS1, CMS2, and CMS3 can be used as oral care probiotics, owing to their inhibitory effect on biofilm formation and volatile sulfur compound formation (1, 2, 8). Here, we report the complete genome sequences of these four strains, which were isolated from the saliva of an infant living in Gwangju, South Korea.

Genomic DNA was extracted from a single colony of each *W. cibaria* strain using the Wizard Genomic DNA purification kit (Promega, Madison, WI, USA). Each strain was sequenced by PacBio (Pacific Biosciences, Inc., Menlo Park, CA, USA) technology using single-molecule real-time (SMRT) analysis version 2.3 software. The PacBio reads were assembled with the HGAP3 protocol and error corrected by Quiver version 1, performed through SMRTpipe version 2.3.0.139497. After correction, Prokka (9) was used for gene prediction and annotation. For additional annotation, the corrected sequences were searched against the GenBank nonredundant (NR) database (downloaded on 27 July 2015) using BLASTx version 2.4.0+ (10).

The complete genome of CMU comprised a circular chromosome of 2,362,501 bp (G+C content of 45.25%) and two circular plasmids of 18,967 bp and 3,467 bp (G+C contents of 38.4% and 50.9%, respectively). CMS1 and CMS2 had circular chromosomal genomes of 2,342,849 bp (G+C content of 45.3%) and 2,342,914 bp (G+C content of 45.3%), respectively. CMS3 had a circular chromosomal genome of 2,342,907 bp (G+C content of 45.24%) and one circular plasmid of 20,585 bp (G+C content of 38.3%). The chromosomal genome of CMU contains 2,038 predicted protein-coding sequences, 33 rRNAs, and 90 tRNAs, with an average gene length of 2,201 bp. The plasmids of CMU contain 21 and 2 predicted protein-coding sequences, with average gene lengths of 25 bp and 2 bp, respectively. The predicted protein-coding sequences of CMS1, CMS2, and CMS3 were 2,024 bp, 2,035 bp, and 2,035 bp, respectively, and the average gene lengths were 2,176 bp, 2,188 bp, and 2,187 bp, respectively. The plasmid of CMS3 contains 23 predicted protein-coding sequences, with an average gene length of 25 bp. The numbers of rRNAs and tRNAs—28 and 88, respectively—were the same for CMS1, CMS2, and CMS3.

### Accession number(s).

The complete genome sequences of the *W. cibaria* strains reported here have been deposited in GenBank under accession no. CP013936 (CMU), CP022606 (CMS1), CP013726 (CMS2), and CP013934 (CMS3).
